# Surgical outcome after standard abdominoperineal resection: A 15-year cohort study from a single cancer centre

**DOI:** 10.1016/j.amsu.2018.10.029

**Published:** 2018-10-31

**Authors:** S. Wilkins, R. Yap, K. Loon, M. Staples, K. Oliva, B. Ruggiero, P. McMurrick, P. Carne

**Affiliations:** a Cabrini Monash University Department of Surgery, Cabrini Hospital, Malvern, VIC, Australia; b Department of Epidemiology and Preventive Medicine, School of Public Health and Preventive Medicine, Monash University, Melbourne, VIC, Australia; c Monash Department of Clinical Epidemiology, Cabrini Hospital, Malvern, VIC, Australia; d Colorectal Unit, Department of Surgery, Alfred Hospital, Melbourne, VIC, Australia

**Keywords:** Colorectal, Surgery, Abdominoperineal, Outcomes

## Abstract

**Background:**

Abdominoperineal resection (APR) is associated with a poorer oncological outcome than anterior resection. This may be due to higher rates of intra-operative perforation and circumferential resection margin involvement. The aim of this study was to audit our short and long-term results of abdominoperineal resection performed using conventional techniques and to compare this with other published series.

**Materials and methods:**

A retrospective review of all patients who had standard APR between January 2000 and December 2016 in a single institution, Cabrini Hospital, Melbourne, Australia. A total of 163 cases performed by nine different colorectal surgeons for primary rectal adenocarcinoma were identified, with their clinicopathological data analysed.

**Results:**

Using standard APR, only six patients (3.7%) were found to have a positive circumferential resection margin (CRM). There were two cases of intra-operative perforation (1.2%). Local recurrence rate was 5.6% of patients, with distant recurrence found in 24.9%. Disease-free survival at five years was 73.1%. Five-year overall survival was 66.7%, 67.9% of all deaths were cancer-related.

**Conclusion:**

Short and long-term outcomes after standard APR in this study were comparable to previous published studies. The CRM rate of 3.7% compares favourably to published positive CRM rates for standard APR which ranged from 6 to 18%. Standard APR remains a viable technique for the treatment of rectal cancer. Patient selection and adequate training remain important factors.

## Highlights

• Retrospective study of APR for rectal cancer in a private hospital over 15 years.
• 163 consecutive cases of abdominoperineal resection 2000–2016.
• 3.7% (6 patients) had a positive circumferential resection margin.
• Local recurrence rate was 5.9%, disease free survival at five years 73.1%.
• Standard APR remains a viable technique for rectal cancer treatment.

## 1 Introduction

Abdominoperineal resection (APR) for treatment of tumours in the mid and lower rectum was first described by Sir Ernest Miles more than 100 years ago and remains a necessary part of rectal oncological surgery in appropriately selected cases [1,2]. The original APR as described by Miles involved completing the perineal component with the patient in the left lateral position [1]. The advent of adjustable leg rests enabled completion of the procedure in the lithotomy position, eliminating the need for repositioning. In 1938, Lloyd-Davies popularised the lithotomy-Trendelenburg position, which was widely adopted for the remainder of the century [3]. Another important concept was one of total mesorectal excision (TME) popularised by Heald in 1982, which has led to lower local recurrence rates in rectal cancer in general [4].

Over the last 30 years, there have been a number of changes in the management of rectal cancers with a resultant decline in the number of patients treated by APR [5,6]. Sphincter preservation, by anterior resection and anastomosis rather than APR has become possible even with low-rectal tumours as a result of technological advances in stapling techniques and in neoadjuvant therapy [2,7]. Although there has been a progressive decline in the number of APRs performed, it remains necessary in certain circumstances, for example, in patients with advanced low rectal tumours where sphincter preservation is impossible.

In addition to the requirement of a permanent stoma, APR is considered a debilitating procedure associated with significant morbidity, particularly in relation to the perineal wound. In recent years, further concerns have emerged with APR as it is associated with a worse oncological outcome compared to anterior resection, with local recurrence rates reported up to 30% in some series, despite aggressive adjuvant therapy [5,8,9].

There are several potential explanations for the poor oncological outcome, mostly related to selection bias. The size of the tumour as reflected by the T staging is an independent prognostic factor in rectal cancer [10]. Patients selected for APR often have large, bulky tumours with more advanced T-stages that are not suitable to sphincter preserving surgery. Wibe et al., also found that lower rectal tumours are more likely to demonstrate poor differentiation compared to tumours in the middle and upper rectum (14% vs. 7%) [8,11]. In cases of borderline sphincter preservation, tumours that respond well to neoadjuvant chemotherapy are also selected towards anterior resections, whereas tumours that do not have the same response undergo APR. This introduces a selection bias in APR patients towards tumours with poorer biological characteristics, and therefore increased rates of local recurrence.

In addition, the experience and training of the surgeon performing the APR is critical [12]. The APR procedure is a technically demanding operation that requires precise dissection to ensure a complete TME. One of the main reasons cited for a poorer oncological outcome in APR is the technical difficulty in achieving a clear circumferential resection margin (CRM) and a higher incidence of intra-operative perforation (IOP) in the lower rectum, both of which are associated with a higher rate of local recurrence [12,13]. The difficulty in obtaining clear CRM and avoiding IOP may be due to an anatomical reduction in protective mesorectal tissue at the pelvic floor with a tendency towards ‘waisting’ of the mesorectum at the level of puborectalis, where the abdominal and perineal approaches typically meet [[14], [15], [16]].

On balance, whilst the long-term outcome of standard APR has been consistently shown to be worse than for anterior resection in many studies, the absolute rate of CRM involvement appears to vary considerably with some series reporting that up to 50% of patients undergoing APR have a positive CRM. These high rates of CRM involvement appear to contradict our own local observations where CRM involvement is an uncommon event. The aim of this study was to audit the CRM involvement, local perforation rate and oncological outcomes at our institution where standard APR is performed, and to compare our results with other published series.

## 2 Material and methods

A retrospective review (cohort study) was performed of all patients who had standard APR between January 2000 and January 2016 in a single tertiary institution, Cabrini Hospital, Melbourne, Australia. Data between 2000 and 2009 was collected retrospectively by chart review and entered into a customized database. In addition to rates of CRM involvement and IOP, other data collected included pathological tumour stage, lymph node yield, presence of lymphovascular invasion, use of neoadjuvant treatment, perioperative mortality rate, and follow-up. Data collected 2010–2015 was prospectively entered into the Cabrini Monash University Department of Surgery colorectal neoplasia database [17]. Ethics approval for the research project was obtained from the Cabrini Human Ethics Research Committee (#02-21-07-14).

Surgical technique was consistent throughout the study period as patients underwent high ligation of the inferior mesenteric artery and pelvic dissection in the TME plane to the level of levator ani. The perineal component was then completed by the same operating surgeon in either the lithotomy or left lateral position. The coccyx was not routinely resected with the pelvic cavity being entered just anterior to the coccyx. An extralevator approach was not used with division of the levator ani occurring from posterior to anterior in close proximity to the external anal sphincter. The perineal wound was closed primarily. No patient in the study required myocutaneous flap or mesh closure of the perineal defect. All operations were carried out by surgeons with post-fellowship colorectal subspecialty training, and who participate in a quality assurance loop, including participation in prospective data entry and regular quality outcome meetings.

A standard histopathological evaluation was performed by an experienced gastrointestinal pathologist with a specialist interest in colorectal cancer. Specimens were serially sliced through the cross-sectional axis and analysed. CRM involvement was defined as the presence of tumour cells within 1 mm of the marked circumferential margin on the final pathology specimen. An intra-operative perforation was defined as a defect in the rectal lumen and accounted for by the pathologist if not detected at time of surgery.

Follow-up occurred with the operating surgeon and included serial clinical, biochemical (with serum carcinoembryonic antigen) and radiological assessment (with computed tomography (CT) scans of the chest/abdomen/pelvis). Typically, patients underwent repeat colonoscopy 12 months post-operatively through the stoma, with further colonoscopy intervals determined by the presence or absence of polyps.

Data were analysed with univariate and multivariate statistical tests (Stata 13, StataCorp LP, College Station, TX, USA). A *P* value of <0.05 was considered significant.

This study has been reported in line with the STROCCS criteria [18]. The research registry unique identifying number for this study is #3786 (www.researchregistry.com).

## 3 Results

One hundred and ninety patients had APR procedures performed between 2000 and 2016 at Cabrini Hospital. Of these cases, 167 were performed for primary rectal adenocarcinoma. The remainder were performed for recurrent adenocarcinoma after previous anterior resection (8), squamous cell carcinoma (6), adenoma (4), ulcerative colitis (3), Crohn's disease (1), or anorectal melanoma (1) and were excluded from further analysis. In four patients, it was clear during peri-operative staging that R0 could not be achieved, and surgery was performed for symptomatic control only, and therefore excluded from the final analysis.

The median age of patients at the time of surgery was 70 years (range 41–95) and 64.4% (105 of 163) were male. Neoadjuvant treatment was received by 83 patients (50.9%). The majority of these (75 of 83, 90.4%) were treated with combined long course chemoradiotherapy and the remainder received preoperative short course radiotherapy. Demographic and clinicopathological data is summarised in Table 1. One hundred and sixty-two patients had elective surgery with only one patient requiring urgent surgery (urgent defined as an operation carried out as soon as possible after resuscitation). This particular patient was an acute presentation with locally advanced disease associated with pain, bleeding and partial obstruction. 27.6% of patients were operated in the lateral position for the perineal phase with the last lateral position patient in 2010. The remaining 72.4% were in the Lloyd-Davies position in the perineal phase. Adjacent structures were resected in 24 patients, with the majority (15/24) being partial or full vaginectomy or oophrectomy.

**Table 1:** Patient demographics and clinicopathologic characteristics.

	APR n = 163	%
Age		
Median (years, range)	70 (41–95)	
Gender		
Male	105	64.4
Female	58	35.6
AJCC Preoperative stage		
I	41	25.2
II	55	33.7
III	47	28.9
IV	13	8.0
Unknown	7	4.3
Neoadjuvant therapy		
SCRT	8	4.9
LCCRT	75	46.0
None	79	48.5
Other	1	0.6
Surgical Entry		
Open	135	82.8
Conversion	5	3.1
Laparoscopic	8	4.9
Robotic	10	6.1
Hybrid	5	3.1
AJCC Pathological Stage		
0	18	11.0
I	43	26.4
II	45	27.6
III	33	20.3
IV	15	9.2
Lymphovascular Invasion		
Yes	123	75.5
No	40	24.5
Differentiation		
Well	5	3.1
Moderate	115	70.5
Poor	21	12.9
No residual	21	12.9
Unknown	1	0.6
Adjacent structures resected	24	14.7
Positive CRM	6	3.7

AJCC, American Joint Committee on Cancer; APR, abdominoperineal resection; CRM, circumferential radial margin; LCCRT, long course chemoradiotherapy; SCRT, short course radiotherapy.

**Fig. 1:**
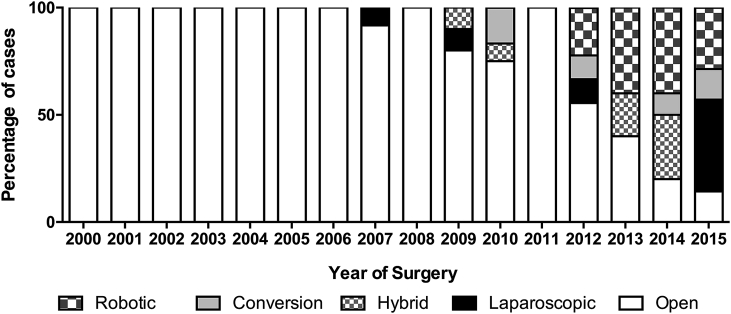
Change in surgical technique over time.

Post-operative pathology showed that cases were spread across all stages; Stage 0 (ypT0N0) 11%, Stage 1 26.4%, Stage 2 27.6%, Stage 3 25.8%, and Stage 4 9.2%. The lymph node yield median was 10 (range 0–55), mean 11.5. The pathological positive CRM rate was 3.7% (6/163). There were two cases of IOP giving an overall intra-operative perforation rate of 1.2%. The majority of patients underwent laparotomy or open surgery (82.8%), whereas other surgical techniques included robotic (6.1%), laparoscopic (4.9%), conversion from laparoscopic to open (3.1%), and hybrid (3.1%; defined as a laparoscopic procedure where an incision is made larger than that simply to retrieve the operative specimen, and some of the dissection done in an open approach). The positive CRM rates for open surgery and minimally invasive surgery were 2.9% (4/135) and 7.1% (2/28) respectively. The IOP rates for open surgery and minimally invasive surgery were 1.4% (2/135) and 0% respectively. The change in the method of surgical entry over the entire study period is shown in Fig. 1.

The 30-day mortality over the entire series (2000–2015) was 1.2%. Over the last five years where database data was available, 30-day mortality was 0%, inpatient death 0%, wound complications 12.2% (6/49), return to theatre 12.2% (the majority for wound related reasons; 6/49), and median length of stay was 10 days, (range 7–28).

**Fig. 2:**
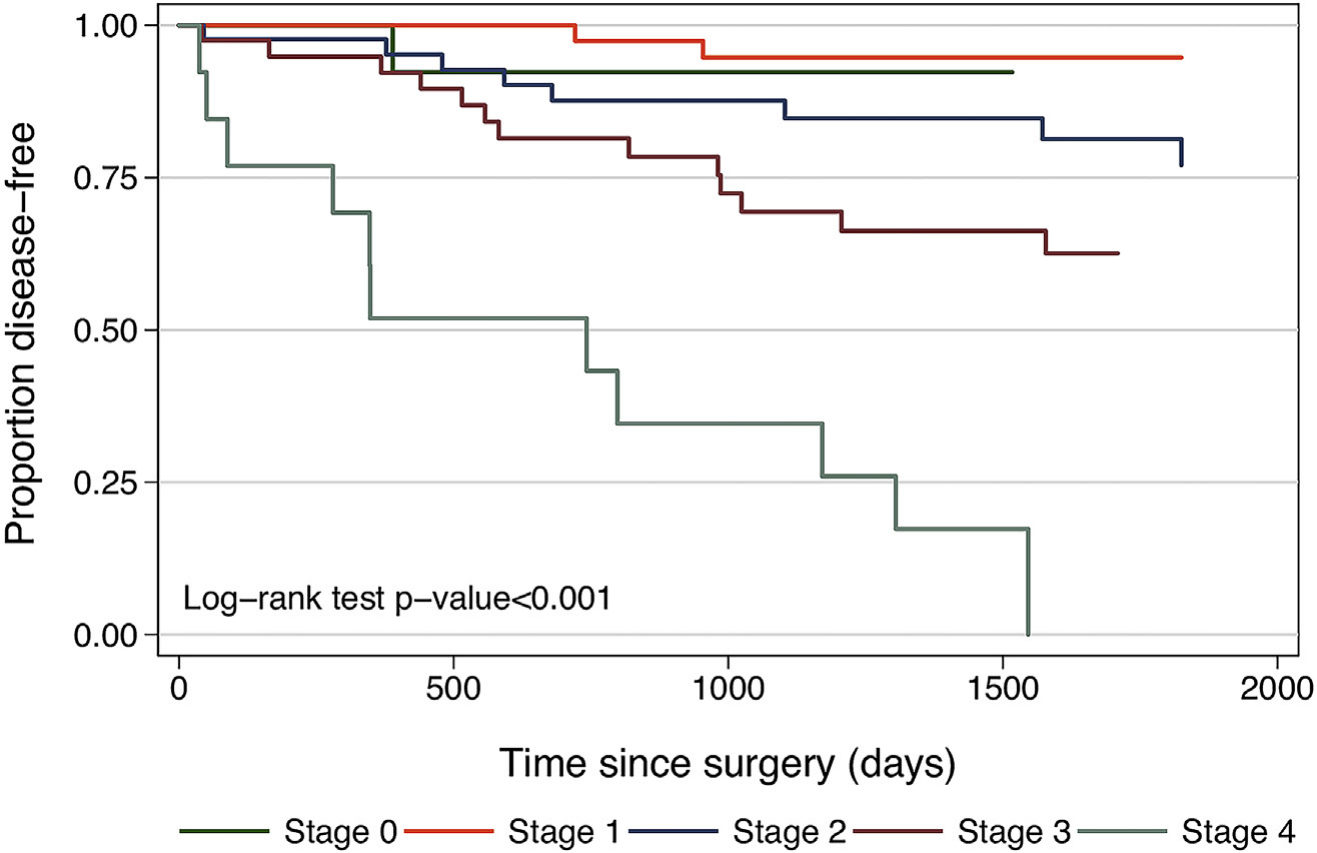
Long term outcomes: Disease-free survival by pathological stage.

Five-year follow-up data was complete for 129 (79.1%) patients. The remaining patients were resected between 2011 and 2016 and therefore, five-year follow-up data was not yet complete at the time of writing. Median follow up time was 52.7 months (range 0.6–142.5 months). Disease-free survival by pathological stage is shown in Fig. 2. Multivariate analysis of factors affecting disease-free outcomes showed an association between increasing stage and poorer outcomes (Table 2). Age-adjusted patients with Stage 4 pathology showed poorer disease-free survival (HR 11.035, 95% CI 1.422–85.666. p = 0.022) (Table 2). Poor disease-free survival was correlated with a positive circumferential resection margin (HR 3.163, 95% CI 1.048–9.549, p = 0.041), the presence of lymphovascular invasion (LVI) (HR 3.764, 95% CI 1.882–7.524, p < 0.001), and a lower lymph node (LN) yield (HR 0.92, 95% CI 0.863–0.981, p = 0.011). In this study, local recurrence rate was 5.6% with a distant metastasis rate of 24.9%. Disease-free survival over five years was 73.1%.

**Table 2:** Multivariate analysis of the factors affecting disease-free survival.

Variable	HR	SE	P	95% CI	
pAJCC					
0	Reference group				
1	0.535	0.662	0.613	0.048	6.034
2	2.078	2.230	0.496	0.254	17.030
3	5.522	5.739	0.100	0.720	42.336
4	11.035	11.539	0.022	1.422	85.666

CRM + status	3.163	1.783	0.041	1.048	9.549
LVI	3.764	1.330	<0.001	1.882	7.524
LN yield	0.920	0.030	0.011	0.863	0.981

CI, confidence interval; CRM, circumferential radial margin; HR, hazard ratio; LN, lymph node; LVI, lymphovascular invasion; pAJCC, pathological stage according to American Joint Committee on Cancer; SE, standard error.

**Fig. 3:**
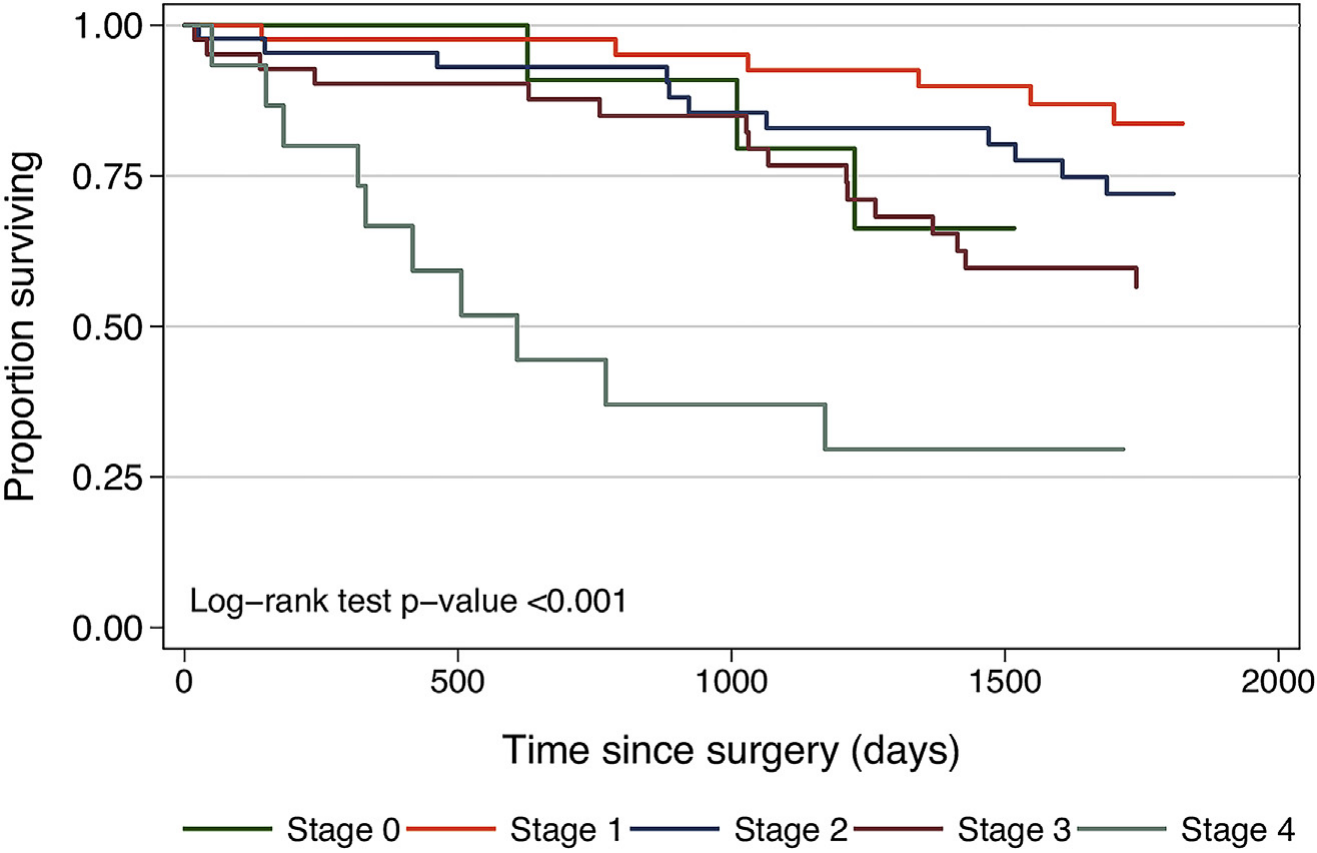
Long term outcomes: Overall survival by pathological stage.

Overall survival by pathological stage is shown in Fig. 3. Multivariate analysis of factors affecting overall survival adjusted for age is shown in Table 3. LVI and pathological staging American Joint Committee on Cancer (pAJCC) showed a strong correlation (Pearson correlation coefficient = 17.93, p = 0.001) meaning that as stage increased, patients were far more likely to have LVI. The correlation was such that LVI and pAJCC could not be used together as independent predictor variables. When adjusted for age, patients with Stage 4 pathology demonstrated poorer outcomes (HR 4.28, 95% CI 1.158–15.819. p = 0.029) (Table 3). When adjusted for age, a correlation was observed between increasing pAJCC pathological stage and poorer outcomes (HR 1.728, 95% CI 1.281–2.331, p < 0.001). Age adjusted LVI status showed that the presence of LVI correlated with poorer outcomes (HR 1.99, 95% CI 1.04–3.809, p = 0.038). Five-year overall survival was 66.7%. Eighteen patients (11%, 18/163) died due to causes unrelated to cancer, accounting for 32.1% of all deaths (18/56).

**Table 3:** Age adjusted multi-variate analysis of factors affecting overall survival.

Variable	HR	SE	P	95% CI	
Age	1.052	0.016	0.001	1.020	1.084
pAJCC					
0	Reference group				
1	0.494	0.355	0.327	0.120	2.025
2	0.827	0.551	0.776	0.224	3.055
3	1.510	0.962	0.517	0.434	5.262
4	4.280	2.855	0.029	1.158	15.819

Age	1.044	0.015	0.003	1.015	1.073
pAJCC	1.728	0.264	<0.001	1.281	2.331

Age	1.040	0.014	0.004	1.013	1.069
LVI	1.990	0.659	0.038	1.040	3.809

CI, confidence interval; HR, hazard ratio; LVI, lymphovascular invasion; pAJCC, pathological stage according to American Joint Committee on Cancer; SE, standard error.

## 4 Discussion

Our data demonstrates that appropriately trained specialist colorectal surgeons using a contemporary technique can safely perform abdominoperineal resection to a high and safe standard. The use of intra-operative perforation rates and CRM involvement as surrogate markers of the oncological adequacy of surgery has previously been described [8,12,19]. The implications of intra-operative perforation and CRM involvement are an increase in local recurrence and poorer oncologic outcomes [20,21]. In our series of 163 patients, using standard APR. we demonstrated a CRM involvement of 3.7% (6 cases) and an intra-operative perforation rate of 1.2% (2 cases). These numbers are lower than many previous published series of patients using standard APR.

Short- and long-term outcomes from this patient cohort were favorable compared with published studies. Patients had 12.2% perineal wound complications (2010–2015 data) comparable to 12% and 28% in two separate Swedish studies [22,23]. The local recurrence rate in this study was 5.6%. Previous studies have shown local recurrence rates of 6.0%, 6.3%, 10.6% and 19.7% following standard APR [[19], [20], [21],^24^]. In this study, poor disease-free survival was correlated with a positive CRM, the presence of LVI, and a lower LN yield. The distant metastasis rate in this patient cohort was 24.9%. Previous studies have shown various rates from 11.6% to 19.9% for standard APR [20,24]. The 5-year disease-free survival was 73.1% that was similar to a Canadian single centre study of 67.4% [21]. The five-year overall survival in this study was 66.7%. Published rates following standard APR have been reported this to be in the range of 59.5%–64.1% [19,24]. Analysis of five randomised clinical trials on rectal cancer showed that OS was associated with LN metastasis and CRM involvement [19].

The main focus of this study was to audit our patients and investigate the CRM+ and IOP in standard APR due to the historically high rates of CRM+ and IOP. Table 4 summarizes the CRM + rates in larger published studies (greater than 50 patients). The recorded CRM + rate following standard APR in this study was 3.7% that compares favourably to many different studies, both multi-centre and single site, in USA and Europe. The IOP rate in this study was 1.2% which is also very favorable compared to many studies reporting rates such as 6.1%, 10.1%, and 11% [21,23,24].

**Table 4:** Observed circumferential positive margin rates in patients undergoing std. APR.

Study	Years	Patients (n)	CRM +
This study^a^	2000–2015	163	3.7%
Prytz et al., 2014 [23]	2007–2009	207	6.3%
Klein et al., 2015 [27]	2009–2012	251	7.2%
den Dulk et al., 2009 [19]	1987–2002	897	10.6%
Ortiz et al., 2014 [28]	2008–2013	457	13.1%
Kennelly et al., 2013 [20]	1990–2011	327	13.9%
Messenger et al., 2011^a^ [21]	1997–2006	115	15.7%
Ortiz et al., 2014 [24]	2006–2010	920	18.1%
Asplund et al., 2012^a^ [22]	2004–2009	75	20.0%
West et al., 2010^a^ [14]	1997–2008	124	49.6%

*Stages 1–3.

a Single centre study. Studies with more than 50 patients.

Some studies have showed poor outcomes using standard APR, with CRM involvement rates of up to 49.6%, intra-operative perforation rates of 28.2% and local recurrence rates of 17.9% [14]. These are in stark contrast with our findings in this study. A positive CRM rate of 49.6% is an outlier rather than being consistent with best practice (Table 4). In West et al., standard APR cases were collected from one institution (Leeds General Infirmary) from 1997 to 2008 [14]. This was a retrospective study of 124 patients by examining photos of the surgical sample. Eight surgeons carried out 2–35 procedures with five specialists performing the majority of cases. Three of these surgeons had combined CRM involvement rates of 39% and IOP of 19% [14]. It is possible that the high rate of 49.6% could be due to sub-optimal surgical technique.

In order to minimise CRM involvement and rates of IOP, several centres in Europe have advocated a change in technique from a standard APR to a more radical extra-levator abdominoperineal excision (ELAPE) [8,14,15,25,26]. There has been much discussion in the literature about the advantages and disadvantages in the ELAPE and standard APR techniques and the oncological outcomes. A Danish study found that CRM involvement rates were higher in ELAPE compared with APR (15.9% vs. 7%) and that ELAPE did not improve short-term oncological outcomes. In this retrospective study, the risk of a positive CRM was higher among ELAPE patients (OR 2.46 95% CI 1.39–4.34, p = 0.002) [27]. This likely reflects a selection bias with larger, more locally advanced tumours being chosen for ELAPE. A large multi-centre study in Spain found that positive CRM rates were almost identical between APR and ELAPE (13.1% vs. 13.6%) [28]. Analysis of the Swedish Colorectal Cancer Registry compared positive CRM and IOP rates for APR and ELAPE. ELAPE had a higher positive CRM rate than APR (10% vs. 6%) whereas IOP rates were higher in APR than ELAPE (11% vs. 8%) [23]. A further Swedish study found positive CRM lower in ELAPE than APR (17% vs. 20%) but higher IOP rates in ELAPE than APR (13% vs. 10%) [22]. There is currently little evidence that routine ELAPE produces better oncological results than APR.

ELAPE is characterised by the increased need for muscle flap or mesh closure (with subsequent complications) resulting in longer operating times and almost double the rate of wound complications from 20% to 38% when compared to APR [14]. In this study where all patients underwent APR, no patients received a flap and the choice of whether to use some form of flap to assist in the closure and healing of the perineal wound was left to the discretion of the operating surgeon. Perineal wound healing was not a primary or secondary outcome measured in this study. It is however recognised that there is significant morbidity associated with perineal wounds, especially following neo-adjuvant chemoradiotherapy.

A middle ground approach tailored to the particular patient and tumour would seem to balance oncological outcomes against the risk of peri-operative complications. The technique used in Silberfein et al., 2010 describes the levator ani being divided widely on the side of the tumour [29]. This is termed neither ELAPE nor standard/conventional APR by George Chang and colleagues at MD Anderson, but a tailored approach depending on the location of the tumour. This approach results in the lowest circumferential positive margin rates described for APR at 1.6% in a cohort of 128 patients. None of the patients with a positive margin developed local recurrence. The 5-year local recurrence rate for the study was 7.9% [29].

Training, rather than technique, may be an important factor in these cases. Patients in centres with higher volumes of rectal work have been shown to have improved outcomes [30,31]. Many of the European centres that have published beneficial outcomes from changing to ELAPE have undergone further training and quality improvement of their results. It may be this process of training that has contributed more to the improvement in results than the technique. Careful patient selection, combined with adequate training, is likely to be the key to improving low rectal cancer oncological outcomes.

The limitations of our study include the retrospective nature of the data from 2000 to 2009 but this is balanced somewhat by the prospective data collected 2010–2015 that was 100% complete with respect to clinician-led data points. This was a single centre study with 163 patients and not a large scale multi-centre investigation of APR. However, a relatively small number of specialist colorectal surgeons at a single centre does however allow for standardisation of technique and surgical quality control.

## 5 Conclusions

In conclusion, given the low CRM+ and IOP rates for standard APR performed by specialist colorectal surgeons over many years at Cabrini Hospital, APR remains a viable technique for the treatment of rectal cancer. The wide variation in surgical quality in terms of CRM+ and IOP in published studies may well be explained by differing standards of surgical training and technique and may also be due to non-specialist colorectal surgeons contributing to study data. A multi-centre Irish study concluded that in specialist units, low CRM + rates are possible with standard APR [20]. Similarly, our results highlight that APR may be performed to a high standard by appropriately trained specialist colorectal surgeons at a single centre. The focus on improving outcomes in rectal cancer should be on standardisation of accepted techniques of APR and importantly, all centres performing APR for rectal cancer should prospectively audit their outcomes, ensure adequate training, conduct careful patient selection and aim at acceptable levels of adverse outcome.

## Provenance and peer review

Not commissioned, externally peer reviewed.

## Conflicts of interest

Nil.

## Funding

This study was funded in part by “Let's Beat Bowel Cancer” (www.letsbeatbowelcancer.com.au), a benevolent fund raising and public awareness foundation.

## Ethical approval

This study was approved. Cabrini Hospital HREC #21-21-07-14.

## Research registration unique identifying number (UIN)

Researchregistry3786.

## Author contribution

1-Study Design.

2-Prospective data collection.

3-Retrospective data collection.

4-Data analysis.

5-Manuscript preparation.

Simon Wilkins (1,2,3,4,5), Raymond Yap (1,2,3,4,5), Kenneth Loon (1,2,3,4,5), Margaret Staples (1,4,5), Karen Oliva (2,3,4,5), Boris Ruggiero (1,2,3,4,5), Paul McMurrick (1,2,3,4,5), Peter Carne (1,2,3,4,5).

## Guarantor

Dr Simon Wilkins.

Mr Raymond Yap.

A/Prof Paul McMurrick.

Mr Peter Carne.

## Appendix A Supplementary data

Wilkins et al the_strocss_statement_checklist Wilkins et al the_strocss_statement_checklist
